# Navigating caregiving challenges: the interplay of health literacy and resilience in families of chronically ill older adults

**DOI:** 10.3389/fpubh.2026.1747125

**Published:** 2026-02-11

**Authors:** Ibrahim Alasqah, Mona Metwally El-Sayed, Ahmed Hashem El-Monshed, Mahmoud Abdelwahab Khedr, Manal Mohammed Hawash, Rahma Mohamed Abdelrahman, Mohamed Hussein Ramadan Atta, Shaimaa Mohamed Amin

**Affiliations:** 1 Department of Community, Psychiatric, and Mental Health Nursing, College of Nursing, Qassim University, Buraydah, Saudi Arabia; 2 Department of Nursing, College of Health and Sport Sciences, University of Bahrain, Manama, Bahrain; 3 Psychiatric and Mental Health Nursing Department, Faculty of Nursing, Alexandria University, Alexandria City, Egypt; 4 Public Health Department, Applied Medical Sciences College, King Khalid University, Khamis Meshait, Saudi Arabia; 5 Family and Community Health Nursing Department, Faculty of Nursing, Damita University, Damita City, Egypt; 6 College of Applied Medical Sciences, Prince Sattam Bin Abdulaziz University, Wadi Addawasir City, Saudi Arabia

**Keywords:** caregivers of older adults, challenges, chronic disease, health literacy, resilience

## Abstract

**Background:**

This study examined the mediating role of resilience in the relationship between health literacy and the challenges faced by caregivers of older adults with chronic diseases in Egypt.

**Methods:**

This study employed a cross-sectional descriptive research design, adhering to the Strengthening the Reporting of Observational Studies in Epidemiology (STROBE) guidelines. Participants were selected through purposive sampling based on specific inclusion criteria, resulting in a sample of 253 caregivers. Data collection involved the Health Literacy Scale for Family Caregivers (HLSFC), the Care Challenge Scale (CCS), and the Resilience Inventory (RESI). The Pearson correlation test examined relationships between continuous parametric data, while linear regression analysis predicted dependent values based on independent variables. Hypothesis testing was conducted using path analysis with the SPSS macro PROCESS.

**Results:**

Significant negative correlations were found between family caregiver health literacy and caregiver challenges (*r* = −0.186, *p* = 0.003) and between health literacy and resilience (*r* = −0.195, *p* = 0.002). A positive correlation was observed between caregiver challenges and resilience (*r* = 0.132, *p* = 0.035). However, resilience did not significantly affect caregiver challenges (B = 0.1388, *p* = 0.1145). The direct effect of health literacy on caregiver challenges was significant (Path C′: B = −0.0619, *p* = 0.0086), as was the total effect (B = −0.0692, *p* = 0.0029). Neither health literacy (B = −0.0901, *p* = 0.7711) nor resilience (B = 0.0454, *p* = 0.9648) was a significant predictor of caregiver challenges in this model. The interaction term (Health Literacy x Resilience) was also insignificant (B = 0.0007, *p* = 0.9273), indicating that resilience does not moderate the relationship between health literacy and caregiver challenges.

**Conclusion:**

This study reveals complex relationships among health literacy, caregiver challenges, and resilience in family caregivers. Higher health literacy is associated with fewer caregiving challenges and lower resilience levels; however, resilience does not mediate or moderate these relationships. Enhancing health literacy among caregivers is crucial for alleviating their challenges and enhancing their caregiving experiences. Future research should focus on targeted interventions to effectively increase health literacy.

## Introduction

The increase in life expectancy and advances in healthcare have contributed to a growing proportion of older adults living with chronic illnesses, placing substantial burdens on healthcare systems and family caregivers (FCs) worldwide. Chronic conditions necessitate long-term management and complex care requirements, particularly in aging populations. Globally, according to the National Council on Aging, nearly 95% of older adults have at least one chronic disease, with almost 80% experiencing comorbidities simultaneously (1, 2). Over 70% of older adults in Egypt live with chronic diseases and have three or more comorbidities (3). Older adults also often face major challenges such as physical limitations, cognitive decline, loneliness, and social isolation, all of which increase their dependence and need for care. This growing prevalence of chronic diseases and challenges not only affects the quality of life (QoL) of older adults but also significantly affects their FCs. These caregivers face many challenges in providing long-term, informal care, including physical, emotional, and financial strain, as well as limited training and support. Furthermore, cultural expectations that family members, particularly women, assume primary responsibility for providing unpaid, long-term care to aging relatives can increase stress and further reduce their QoL (4, 5). These challenges are particularly pronounced in the Middle East and North Africa (MENA) region, including Egypt, where rising rates of chronic disease are driven by an aging population and lifestyle changes, which are worsened by limited healthcare resources, thereby increasing FCs’ burdens (4–6). Consequently, FCs in MENA face increased demands and challenges in managing complex, long-term care within an often-limited healthcare infrastructure and insufficient access to training, counseling, and financial support (4, 5).

Caregiving is inherently complex and requires a multifaceted approach to address the diverse and evolving needs of older adults. It is also profoundly ingrained in Egyptian society, where social expectations place a central role on family members to provide primary care for aging relatives (6, 7). Family caregivers often handle tasks such as medication management, mobility assistance, and emotional support. Given the breadth of these responsibilities, this reliance on informal care highlights the importance of support systems that focus on providing education and training for FCs. However, limited healthcare services in Egypt make caregiving for older adults physically and mentally challenging, especially for FCs who often lack psychosocial support (3, 7). Because caregiving duties are relentless, caregivers of chronically ill older adults are at a higher risk of burnout, which can show up as exhaustion, frustration, anxiety, and emotional detachment, affecting their mental health and their ability to provide quality care (8, 9). Family caregivers may also reduce their work hours or leave their jobs to fulfill their caregiving roles, resulting in income loss, increased healthcare expenses, and economic hardship. In Egypt, limited health insurance coverage and social support systems exacerbate the financial and emotional strain on FCs (7, 9). The absence of long-term care policies also shifts almost all responsibility to families, with little formal respite or financial support. The lack of caregiver service integration within healthcare limits access to training, counseling, and community services, leaving caregivers without guidance, which ultimately raises caregiver stress (9, 10). However, certain interventions have been shown to reduce caregiver stress, burden, and challenges, such as receiving consistent educational support from healthcare specialists (11), respite services (12), sharing responsibilities within the family (13), and developing coping strategies through health literacy and resilience training (14, 15).

Health literacy is a multidimensional concept that encompasses motivation, knowledge, and competence required to find, interpret, evaluate, and apply health information to make informed choices in everyday health-related activities, such as promoting health, preventing illness, and managing healthcare. Evidence consistently associates low health literacy with adverse outcomes, including increased hospitalization rates, diminished QoL, and delayed diagnoses. Conversely, high health literacy enables FCs to accurately interpret medical information, adhere effectively to treatment plans, and make well-informed decisions, thereby improving care outcomes (16, 17). A study in Egypt by Rahman found that 75% of FCs had low health literacy significantly linked to worse health outcomes in older adults (18). In response to these challenges, the World Health Organization (WHO) has proposed a conceptual framework that positions health literacy as a key determinant of adaptive capacity, including skills in appraisal and self-care, which promote resilience, reduce health disparities, and enhance overall well-being (19). Building on this foundation, resilience emerges as a crucial psychological resource that helps caregivers manage sustained demands (14). Empirical evidence supports this framework and demonstrates that higher health literacy enhances FCs’ self-care and health management capabilities (20). Furthermore, a randomized clinical trial demonstrated that bolstering adaptive capacity, through Roy’s Adaptation Model, directly enhances resilience and adaptation in caregivers (21). Thus, this study is one of the first in Egypt to examine resilience as a mediator between health literacy and caregiver challenges, underscoring the value of formally testing this mediation pathway. Given Egypt’s limited support infrastructure for caregivers, understanding modifiable factors like health literacy and resilience is essential for developing culturally tailored interventions in resource-constrained settings (18, 22).

A recent perspective on managing FCs’ strain highlights the crucial role of psychological resources, especially resilience, which significantly impacts caregivers’ health and well-being (15). The American Psychological Association (APA) defines resilience as the process and outcome of adapting effectively to challenging situations through mental, emotional, and behavioral flexibility and adjustments (23). Specifically, resilience refers to the ability of FCs to cope with caregiving stressors, enhance their self-care, and manage tasks effectively. Resilient caregivers are able to adapt to changes and recover quickly from stress (24). Research shows that resilience in FCs is linked to lower depression, better health, social support, and improved older adults’ care. It acts as a protective factor, helping manage caregiving burdens, reduce stress, and sustain their roles (24, 25). Ali et al. highlighted a negative link between FCs’ burden and psychological resilience, especially with limited social support and high caregiving demands. Many caregivers with low or moderate resilience face increased risks of burnout, anxiety, and declining caregiving capacity, especially with limited health literacy (26). Therefore, improving resilience through targeted interventions, such as education programs combined with resilience-building training that are tailored to resource-limited settings, could enhance caregivers’ quality of life (15). Beyond health literacy and resilience, caregiver challenges are shaped by various socioeconomic and clinical factors. Gérain and Zech (2019) identified lower socioeconomic status encompassing education, employment, and income as a key risk factor for burden. The type and severity of chronic illness are also strongly predictors of adverse caregiver outcomes (27).

While both health literacy and resilience are recognized as factors influencing caregiving outcomes, limited research has examined resilience as a mediating factor between health literacy and FCs’ challenges (14, 15). This study addresses this critical gap in the Egyptian and MENA literature, where resilience has rarely been examined as a mechanism linking health literacy to caregiver outcomes. By testing this mediation model, the research suggests that resilience is a modifiable factor that can inform cultural and cost-effective interventions aimed at enhancing caregivers’ coping skills and improving the quality of care for older adults. This is one of the first studies in Egypt and among the few in the MENA region to statistically analyze resilience as a mediator in this context, offering crucial evidence to guide targeted support programs and policy initiatives for caregivers in resource-limited settings. In the MENA context, with a specific focus on Egypt, this study examines whether resilience mediates the relationship between health literacy and caregiver burden among family caregivers of older adults with chronic conditions.

### Research hypotheses

1) Caregivers with higher levels of health literacy will experience lower levels of caregiving challenges.
2) Caregivers with higher levels of resilience will experience lower levels of caregiving challenges.
3) Higher caregiver health literacy is positively associated with greater resilience.
4) Resilience mediates the relationship between health literacy and caregiver challenges.

## Methods

### Study design and setting

This study used a cross-sectional descriptive research design, adhering to the Strengthening the Reporting of Observational Studies in Epidemiology (STROBE) guidelines. The study was conducted at health insurance outpatient clinics in Damietta, Egypt. These clinics provide specialized care for chronic health conditions such as diabetes, hypertension, and heart disease, with regular follow-ups to monitor and adjust treatment plans as needed.

### Sample size and study population

The target group for the current study was the caregivers of older adults with chronic diseases. Participants in the study were caregivers aged 18 and older who were responsible for caring for older adults (aged 60 and above) diagnosed with chronic diseases. To ensure substantial experience with caregiving challenges, caregivers must have provided care for at least 6 months. Exclusion criteria included professional healthcare providers, as their experiences and resources differ significantly from those of informal caregivers. Caregivers of older adults with acute or temporary health conditions were also excluded to maintain a focus on chronic disease management.

The determination of our sample size was conducted through a meticulous power analysis using G*Power version 3.1.9.7. This analysis was based on a 95% confidence level, a 5% alpha (significance) level, and a suggested medium effect size of 0.15 (28). Considering the complexity of our regression analysis, which involved 13 predictors encompassing the Health Literacy Scale-Family Caregiver challenges, resilience in FCs of older adults, and 10 demographic variables, the minimum required sample size was calculated to be 189. An anticipated dropout rate of 30% was established to address potential challenges related to our chosen data collection method (29, 30). Thus, we prudently decided to recruit an additional 70 participants. This adjustment increases our sample size, ensuring the robustness, integrity, and reliability of our findings. The larger sample protects against potential data collection issues and enhances the overall generalizability and external validity of the study’s results. Considering these factors, 259 participants were asked to complete the structured interview using questionnaires.

### Sampling method and recruitment

A purposive sampling strategy was employed to recruit participants who met predefined inclusion criteria. Initially, 259 caregivers were invited to participate in the study. Of these, two declined to participate, and four did not meet the eligibility requirements. Consequently, the final analytical sample comprised 253 caregivers whose responses were included in the study.

### Measurement of interests

#### The demographic form

The data collection included information about the caregivers’ age, marital status, level of education, place of residence, occupation, and family income. Additionally, we collected data on the older adults’ age, sex, level of education, type of chronic disease, and duration of chronic disease diagnosis.

#### Health literacy scale family caregiver

The Health Literacy Scale-Family Caregiver (HLSFC), developed by Kor et al., assesses health literacy among FCs of older adults with chronic illnesses (16). It includes 42 items across four levels of health literacy (accessing, understanding, appraising, and applying health information) and five subscales: symptom management (9 items), which measures the ability to recognize, interpret, and respond to health symptoms; daily care (9 items), which evaluates competence in performing routine care tasks such as hygiene and mobility; care coordination (7 items), which gages skill in organizing and communicating with healthcare providers and services; communication (7 items), assessing the ability to discuss health-related information clearly and effectively, and self-care (10 items), which measures awareness of and practices for maintaining the caregiver’s own health and well-being. Items are rated on a four-point Likert scale, from 1 (very difficult) to 4 (very easy), with higher scores indicating greater proficiency in managing caregiving-related health information. The scale demonstrated strong validity and reliability, supported by confirmatory factor analysis that validated the five-factor structure. Concurrent validity was confirmed with the European Health Literacy Questionnaire (*r* = 0.67, *p* < 0.01), and internal consistency was high (Cronbach’s *α* = 0.96), with acceptable test–retest reliability (*r* = 0.77, *p* < 0.01) (16). In the present study, the scale showed strong reliability, with a Cronbach’s alpha of 0.89. After translating the scale into Arabic, exploratory factor analysis confirmed satisfactory content validity, with factors accounting for 78.151% of the variance. The Kaiser-Meyer-Olkin measure and Bartlett’s test of sphericity further supported the scale’s suitability for Arabic-speaking populations.

#### Care challenge scale

The Care Challenge Scale (CCS) was developed by Sharif et al. to assess the challenges faced by FCs (31). The scale consists of 10 items, divided into two subscales: practical role-play challenge (5 items) and lack of social and financial support (5 items). Each item is rated on a five-point Likert scale, ranging from “never” (1) to “always” (5), with higher scores indicating more significant challenges and difficulties that caregivers face. The CCS demonstrated strong validity and reliability. Confirmatory factor analysis confirmed a good fit for the two-factor model, explaining 42.23% of the total variance and establishing the scale’s construct validity through convergent and discriminant validity. Reliability indices showed robust internal consistency, with Cronbach’s alpha values of 0.87 for the influential role-play challenge factor and 0.85 for the lack of social-financial support factor. McDonald’s omega coefficients were 0.88 and 0.86, respectively. The scale also demonstrated stability, as evidenced by a high intraclass correlation coefficient (ICC) of 0.89, indicating its absolute reliability. The scale demonstrated excellent internal consistency in the current study, with a Cronbach’s alpha coefficient of 0.90. After translating the scale into Arabic, the researchers conducted an exploratory factor analysis to assess its validity. Factor loadings ranged from 0.460 to 0.820 before rotation and improved to 0.590 to 0.940 after varimax rotation. All factor loadings exceeded the 0.40 threshold, collectively explaining 70.512% of the total variance.

#### Resilience inventory

The Resilience Inventory (RESI), originally designed and validated by Gaxiola Romero et al. (32), was later validated for use with FCs of older adults by Mandujano et al. (33). The instrument comprises 12 items divided into two dimensions: instrumental resilience (7 items), which assesses practical problem-solving abilities, and emotional resilience (5 items), which focuses on emotional strength and coping strategies. Respondents rate the frequency of specific thoughts, feelings, or behaviors over the past month on a 4-point Likert scale (1 = never, 2 = a few times, 3 = many times, 4 = always). Higher scores indicate greater resilience. According to Mandujano et al., the RESI demonstrated high internal consistency, with a Cronbach’s alpha of 0.93, indicating excellent reliability (33). The scale was validated using exploratory factor analysis (EFA) and confirmatory factor analysis (CFA). The EFA, conducted with 125 caregivers, identified two factors with eigenvalues greater than 1, explaining 64.3% of the total variance. The CFA, performed with a separate sample of 160 caregivers, confirmed the two-factor structure with fit indices as follows: CFI = 0.96, TLI = 0.95, RMSEA = 0.04, and SRMR = 0.03, demonstrating strong construct validity for the RESI. In the current study, the scale showed excellent internal consistency, with a Cronbach’s alpha coefficient of 0.93. After translating the scale into Arabic, researchers conducted an exploratory factor analysis to assess its validity. Factor loadings ranged from 0.50 to 0.85 before rotation and improved to 0.60–0.92 after varimax rotation. All factor loadings exceeded the 0.40 threshold, collectively explaining 68.7% of the total variance.

### Study procedures

#### Tool preparation and pilot study

The research instruments, including HLS-FC, CCS, and RESI, were translated into Arabic with meticulous attention to detail. Bilingual experts, fluent in both English and Arabic, undertook the translation process to ensure accuracy and cultural appropriateness. To validate the translations, a back-translation into English was performed to confirm linguistic equivalence and address any discrepancies that may have arisen. Following the translation and back-translation, face validity assessments were conducted for each instrument. Expert panels reviewed the translated tools to ensure they accurately captured the intended constructs within the Arabic context.

Additionally, feedback was gathered from potential participants to confirm the clarity, relevance, and cultural appropriateness of the translated items. Reliability was assessed using statistical methods, including Cronbach’s alpha, to ensure internal consistency. A pilot study involving 20 caregivers was conducted to evaluate the clarity, relevance, and reliability of the instruments. The participants of this pilot study were subsequently excluded from the main study. The pilot study results indicated no necessary modifications to the tools, affirming their suitability for the primary research.

#### Data collection

The data collection process commenced by providing participants with a comprehensive orientation that outlined the study’s objectives and emphasized the voluntary nature of their participation. Researchers promptly addressed any questions or concerns raised by participants and reiterated the confidentiality measures in place to build trust in the research process. Trained researchers conducted data collection between June and August 2024. Written informed consent was obtained from each participant before they participated in the study. Participants were instructed not to include personal identifiers on the questionnaire to ensure anonymity. Participation was voluntary, and participants could withdraw from the study at any time. The researchers conducted interviews with participants in the waiting area of the health insurance clinics. Each interview lasted between 20 and 30 min and was conducted weekly, from Saturday to Thursday, between 10 a.m. and 2 p.m. This schedule was designed to accommodate the participants’ availability while ensuring thorough data collection.

#### Ethical considerations

Approval was obtained from the Research Ethics Committee of the Faculty of Nursing at Damietta University, Egypt. Additionally, permission was granted by the clinic directors. The study adhered to the ethical principles outlined in the Declaration of Helsinki, thereby protecting the rights and well-being of participants. All participants provided written informed consent following a thorough explanation of the study’s objectives. Participants’ privacy and anonymity were rigorously protected, and all data collected was treated with the utmost confidentiality.

#### Statistical analysis

The Statistical Package for Social Sciences (SPSS), version 29, was used for data analysis. All the categorical data are summarized as percentages and frequencies. Continuous variables are presented as Mean ± Standard Deviation (SD). The Pearson correlation test was used to analyze the correlation between continuous parametric data. Linear regression analysis was used to “predict” the dependent variable’s value based on the predictors’ values. Hypothesis testing involved path analysis using the SPSS macro named PROCESS (34). All statistical tests adhered to the alpha level of 0.05 to determine statistical significance.

## Results

Table 1 reveals that most caregivers in this study were young adults, with slightly over half (51%) falling within the 20- to 29-year age range. In terms of education, nearly half of the caregivers (44.7%) had only primary education. Most caregivers (73.1%) resided in urban areas, while the remaining 26.9% lived in rural regions. The older adults under the care of the participants were predominantly in the 70 years and above age group, comprising 47.4%. Females comprised the majority of the older adult population at 66%, while males accounted for 34%. When looking at the chronic diseases that the older adults suffered from, heart disease was the most common, affecting 32% of the participants. This was followed by hypertension, which affected 28.5%, while 24.1% of the older adults had chronic kidney disease, and 15.4% had diabetes.

**Table 1:** Socio-demographic characteristics of the study participants and their older adults (*N* = 253).

Socio-demographic factors	*N*	%
(I) Caregivers’ data		
Age (in Years)		
20–29	129	51.0
30–39	42	16.6
40–49	53	20.9
≥ 50	29	11.5
Level of education		
Primary education	113	44.7
Secondary education	108	42.7
University and postgraduate	32	12.6
Marital status		
Single	24	9.5
Married	154	60.9
Divorced	31	12.3
Widowed	44	17.3
Residence		
Rural	68	26.9
Urban	185	73.1
Employment status		
Working	171	67.6
Not working	82	32.4
Family income		
Sufficient	42	16.6
Insufficient	77	30.4
Sufficient and save	133	52.6
(II) Older adults’ data		
Age (in Years)		
60–65	81	32.0
65–70	52	20.6
≥ 70	120	47.4
Gender		
Female	167	66.0
Male	86	34.0
Level of education		
Basic education	76	30.0
Secondary education	136	53.8
University and postgraduate	41	16.2
Chronic disease type		
Diabetes	39	15.4
Hypertension	72	28.5
Heart diseases	81	32.0
Chronic kidney diseases	61	24.1
Chronic disease duration (in Years)		
< 5	47	18.6
5–10	112	44.3
≥ 10	94	37.2

As shown in Table 2, the total family caregiver health literacy scores ranged from 78 to 150, with a mean score of 125.78 (SD = 15.36). This indicates that, on average, the caregivers in this study exhibited a relatively high level of health literacy. The total scores for caregiver challenges ranged from 14 to 35, with a mean of 23.99 (SD = 5.69), indicating that caregivers experienced moderate challenges. Within this domain, the *Social Financial Challenges* subdomain had a slightly higher mean (*M* = 12.25, SD = 3.21) than Effective Role Play Challenges (*M* = 11.74, SD = 3.04). The total resilience score among caregivers ranged from 19 to 44, with a mean of 38.44 (SD = 4.10), indicating a relatively high level of overall resilience.

**Table 2:** Descriptive statistics of the study measures (*N* = 253).

Study measures	Minimum	Maximum	*M*	SD
Family caregiver health literacy (Total)	78	150	125.78	15.36
Symptom Management	16	32	26.92	3.61
Daily Care	14	32	26.66	3.84
Care Coordination	11	27	20.65	3.24
Communication	9	27	21.18	3.17
Self-Care	15	37	30.36	4.36
Caregiver challenges (Total)	14	35	23.99	5.69
Effective Role Play Challenges	6	19	11.7391	3.04
Social Financial Challenges	6	19	12.2530	3.21
Resilience (Total)	19	44	38.44	4.10
Instrumental Resilience	10	26	21.9526	2.76
Emotional Resilience	8	19	16.4862	1.86

M, Mean; SD, Standard Deviation; Symptom Management: 9–36; Daily Care: 9–36; Care Coordination: 7–28; Communication: 7–28; Self-Care: 10–40.

The correlation analysis presented in Table 3 highlights the relationships between the total scores of the study’s key variables: family caregiver health literacy, caregiver challenges, and resilience. A significant negative correlation was found between family caregiver health literacy and caregiver challenges (*r* = −0.186, *p* = 0.003). Moreover, a significant negative correlation exists between family caregiver health literacy and resilience (*r* = −0.195, *p* = 0.002). A significant positive correlation was also observed between caregiver challenges and resilience (*r* = 0.132, *p* = 0.035).

**Table 3:** Correlation coefficient analysis between the study measures (*N* = 253).

Study variables		(1)	(2)	(3)	(4)	(5)	(6)	(7)	(8)	(9)	(10)	(11)	(12)
Family caregiver health literacy (1)	*r*	1											
	*P*												
Symptom management (2)	*r*	0.824^**^	1										
	*P*	<0.001											
Daily care (3)	*r*	0.871^**^	0.696^**^	1									
	*P*	<0.001	<0.001										
Care coordination (4)	*r*	0.816^**^	0.593^**^	0.667^**^	1								
	*P*	<0.001	<0.001	<0.001									
Communication (5)	*r*	0.845^**^	0.564^**^	0.656^**^	0.675^**^	1							
	*P*	<0.001	<0.001	<0.001	<0.001								
Self-care (6)	*r*	0.850^**^	0.611^**^	0.637^**^	0.561^**^	0.699^**^	1						
	*P*	<0.001	<0.001	<0.001	<0.001	<0.001							
Caregiver challenges (7)	*r*	−0.186^**^	−0.157^*^	−0.130^*^	−0.144^*^	−0.172^**^	−0.179^**^	1					
	*P*	0.003	0.012	0.039	0.022	0.006	0.004						
Effective role play challenges (8)	*r*	−0.212^**^	−0.172^**^	−0.148^*^	−0.170^**^	−0.184^**^	−0.212^**^	0.904^**^	1				
	*P*	<0.001	0.006	0.019	0.007	0.003	<0.001	<0.001					
Social financial challenges (9)	*r*	−0.130^*^	−0.116	−0.090	−0.093	−0.131^*^	−0.117	0.915^**^	0.654^**^	1			
	*P*	0.039	0.065	0.153	0.140	0.038	0.063	<0.001	<0.001				
Resilience (10)	*r*	−0.195^**^	−0.114	−0.165^**^	−0.208^**^	−0.190^**^	−0.155^*^	0.132^*^	0.164^**^	0.079	1		
	*P*	0.002	0.070	0.009	<0.001	0.002	0.013	0.035	0.009	0.212			
Instrumental resilience (11)	*r*	−0.151^*^	−0.099	−0.121	−0.176^**^	−0.152^*^	−0.101	0.099	0.135^*^	0.048	0.925^**^	1	
	*P*	0.016	0.114	0.054	0.005	0.016	0.109	0.115	0.031	0.449	<0.001		
Emotional resilience (12)	*r*	−0.205^**^	−0.103	−0.183^**^	−0.197^**^	−0.191^**^	−0.191^**^	0.144^*^	0.161^*^	0.102	0.827^**^	0.551^**^	1
	*P*	0.001	0.102	0.004	0.002	0.002	0.002	0.022	0.010	0.105	<0.001	<0.001	

r, Pearson correlation. **Correlation is significant at the 0.01 level (2-tailed). *Correlation is significant at the 0.05 level (2-tailed).

The regression analysis in Table 4 reveals that several socio-demographic factors, health literacy, and resilience are significant predictors of caregiver challenges, with an adjusted *R*^2^ of 0.775. The caregiver’s age approached significance (*ß* = 0.080, *p* = 0.056). In contrast, educational level (*ß* = −0.071, *p* = 0.026), employment status (*ß* = −0.077, *p* = 0.015), family income (*ß* = −0.303, *p* < 0.001), older adult’s age (*ß* = 0.182, *p* < 0.001), chronic disease type (*ß* = 0.511, *p* < 0.001), health literacy (*ß* = −0.077, *p* = 0.017), and resilience (*ß* = 0.082, *p* = 0.009) were all significant predictors of caregiver challenges. Chronic disease duration was not a significant predictor (*β* = 0.048, *p* = 0.124).

**Table 4:** Regression coefficients: Socio-demographic data, health literacy, and resilience as predictors of caregiver challenges.

Predictors	Caregiver challenges						
	Unstandardized coefficients		Standardized coefficients	*t*	*p*	95% CI for difference	
	*B*	SE	*ß*			Lower bound	Upper bound
Constant	18.170	3.012		6.032	<0.001	12.236	24.103
Caregiver’s age	0.423	0.220	0.080	1.924	0.056	−0.010	0.857
Caregiver’s educational level	−0.586	0.262	−0.071	−2.235	0.026	−1.102	−0.069
Caregiver’s employment status	−0.942	0.384	−0.077	−2.454	0.015	−1.699	−0.186
Family income	−2.302	0.280	−0.303	−8.216	<0.001	−2.854	−1.750
Older adults’ age	1.178	0.227	0.182	5.182	<0.001	0.730	1.625
Older adults’ gender	0.683	0.376	0.057	1.817	0.071	−0.058	1.423
Chronic disease type	2.877	0.277	0.511	10.382	<0.001	2.331	3.423
Chronic disease duration	0.378	0.245	0.048	1.544	0.124	−0.104	0.861
Health Literacy	−0.028	0.012	−0.077	−2.402	0.017	−0.052	−0.005
Resilience	0.114	0.043	0.082	2.640	0.009	0.029	0.198

Effect (B), Unstandardized coefficient; ß, standardized coefficient beta; SE, Standard Error; *t*, Student *t* test for linear regression. 95% CI for difference 95% Confidence Interval for Difference. *R*^2^ = 0.784 (Adj R^2^ = 0.775), F-change = 87.504, *p*, significance level. Dependent variable: Caregiver Challenges.

The mediation analysis in Table 5 examines the effect of health literacy on caregiver challenges through the lens of resilience. Path A shows a significant negative impact of health literacy on resilience (*B* = −0.0522, *p* = 0.0018), indicating that higher health literacy is associated with lower resilience. However, Path B, which assesses the effect of resilience on caregiver challenges, is insignificant (*B* = 0.1388, *p* = 0.1145). The direct impact of health literacy on caregiver challenges (Path C′) is significant (B -0.0619, *p* = 0.0086), while the total effect of health literacy on caregiver challenges is also significant (*B* = −0.0692, *p* = 0.0029). The indirect effect of health literacy on caregiver challenges through resilience is not statistically significant, as the bootstrapped 95% confidence interval includes zero [−0.0172, 0.0010]. Thus, resilience does not significantly mediate the relationship between health literacy and caregiver challenges.

**Table 5:** Mediation analysis of the effect of health literacy on caregiver challenges through resilience.

Path	Effect (B)	SE	*t*	*p*	95% CI for difference	
					Lower bound	Upper bound
A: (HL → Res)	−0.0522	0.0165	−3.157	0.0018	−0.0847	−0.0196
B: (Res → CC)	0.1388	0.0876	1.584	0.1145	−0.0338	0.3113
C: (HL → CC) Direct Effect	−0.0619	0.0234	−2.648	0.0086	−0.1080	−0.0159
C: (HL → CC) Total Effect	−0.0692	0.0230	−3.007	0.0029	−0.1145	−0.0239
Indirect Effect (Path A × Path B)	−0.0072	0.0047	–	–	−0.0172	0.0010

HL, Health Literacy; Res, Resilience; CC, Caregiver Challenges. Effect (B), Unstandardized Coefficient; *ß*, standardized coefficient beta; SE, Standard Error; *t*, Student *t* test for linear regression; *p*, significance level; 95% CI for difference = 95% Confidence Interval for Difference. Model Summary for Resilience (Path A): *R*^2^ = 0.0382, F (1, 251) = 9.9648, *p* = 0.0018. Model Summary for Caregiver Challenges (Path B and Path C): *R*^2^ = 0.0443, *F* (2, 250) = 5.8009, *p* = 0.0034. Bootstrap Confidence Interval for Indirect Effect: Bootstrapped 95% CI: [−0.0172, 0.0010] (Not statistically significant). Path A: The effect of Health Literacy on Resilience. Path B: The Effect of Resilience on Caregiver Challenges. Path C′: The direct effect of Health Literacy on Caregiver Challenges (after accounting for Resilience). Total Effect (Path C): The overall effect of health literacy on caregiver challenges. Indirect Effect: The effect of health literacy on caregiver challenges.

The moderation analysis, as illustrated in Table 6, investigates whether resilience moderates the relationship between health literacy and caregiver challenges. The results show that neither health literacy (*B* = −0.0901, *p* = 0.7711) nor resilience (*B* = 0.0454, *p* = 0.9648) significantly predicts caregiver challenges in this model. Additionally, the interaction term (Health Literacy × Resilience) is insignificant (*B* = 0.0007, *p* = 0.9273), indicating that resilience does not moderate the relationship between health literacy and caregiver challenges. The overall model is statistically significant (*R*^2^ = 0.0444, *F* = 3.8547, *p* = 0.0101), but the interaction effect does not contribute significantly to the explained variance (*R*^2^ Change = 0.000, *F* = 0.0083, *p* = 0.9273). Thus, resilience does not play a moderating role in the relationship between health literacy and caregiver challenges.

**Table 6:** Moderation analysis of resilience in the relationship between health literacy and caregiver challenges.

Coefficients	Effect (B)	SE	*ß*	*t*	*p*	95% CI for difference	
						Lower bound	Upper bound
Constant	30.1265	40.6106		0.7418	0.4589	−49.8576	110.1106
Health literacy	−0.0901	0.3092	−0.2913	−0.2913	0.7711	−0.6991	0.5189
Resilience	0.0454	1.0265	0.0442	0.0442	0.9648	−1.9763	2.0671
Interaction term (Health Literacy x Resilience)	0.0007	0.0078	0.0913	0.0913	0.9273	−0.0147	0.0161

Model Summary: *R*^2^ = 0.0444, *F* = 3.8547, *p* (Model) = 0.0.0101. Test of Interaction Effect: *R*^2^ Change 0.000, F (Interaction) = 0.0083, *p* (Interaction) = 0.9273. Effect (B), Unstandardized Coefficient; *ß*, standardized coefficient beta; SE, Standard Error; *t*, Student *t* test for linear regression; *p*, significance level; 95% CI for difference, 95% Confidence Interval for Difference. Dependent variable: Caregiver challenges.

## Discussion

Caregivers of older adults with chronic diseases play a crucial role in supporting and managing health-related tasks. Yet, they often face significant challenges that can impact their well-being. The study aimed to investigate the mediating role of resilience in the relationship between health literacy and the challenges caregivers face in caring for older adults with chronic diseases.

The current study findings indicate that caregivers generally possess a high level of health literacy, as evidenced by the total scores on the Family Caregiver Health Literacy measure. This suggests that caregivers have the necessary skills to access, understand, and apply health information effectively, which is crucial for managing the complexities associated with chronic disease care. The breakdown of health literacy into specific domains reveals that caregivers excel particularly in symptom management and daily care, which are essential components of caregiving. These high scores reflect the caregivers’ familiarity with their care recipients’ health conditions and ability to navigate the healthcare system effectively. However, the relatively lower score in care coordination indicates potential areas for improvement, displaying that caregivers may face challenges in coordinating care among various healthcare providers and services. This aligns with existing literature highlighting the importance of effective communication and coordination in enhancing caregiver experiences and outcomes (35).

Regarding caregiver challenges, the moderate mean score indicates that while caregivers face difficulties, these challenges are not overwhelmingly high. Notably, the higher mean score in the Social Financial Challenges subdomain compared to the Effective Role Play Challenges denotes that financial and social support issues may be more pressing for caregivers. This finding is consistent with previous research that emphasizes the financial strain often experienced by caregivers, which can exacerbate stress and impact their overall well-being (36, 37). Addressing these financial challenges is crucial, as they can significantly impact caregivers’ ability to provide care and maintain their health.

The resilience scores indicate that caregivers exhibit a relatively high level of resilience, which is a positive finding. Resilience is essential for caregivers as it enables them to cope with caregiving’s emotional and physical demands (38). The distinction between instrumental and emotional resilience underscores that while caregivers may feel equipped to handle the practical aspects of care, there may be room for growth in providing emotional support and developing effective coping strategies. This suggests that interventions to enhance emotional resilience benefit caregivers, enabling them to manage stress more effectively and prevent burnout.

The findings reveal several significant correlations that underscore the complex dynamics of caregiving. Firstly, the negative correlation between family caregiver health literacy and caregiver challenges denotes that higher levels of health literacy are associated with fewer challenges caregivers face. This relationship indicates that caregivers who are more knowledgeable about health-related information and resources may be better equipped to manage the demands of caregiving, thereby experiencing less stress and fewer obstacles in their roles. This aligns with existing literature that emphasizes the importance of health literacy in enhancing caregiver efficacy and reducing the burden associated with caregiving responsibilities (39, 40).

Moreover, the negative correlation between family caregiver health literacy and resilience suggests a nuanced relationship rather than a straightforward protective effect. Caregivers with higher health literacy often have a more detailed understanding of the illness trajectory, treatment complexities, and potential complications, which may heighten perceived burden and emotional strain, thereby undermining their resilience (38). This indicates that caregivers with high health literacy may feel overwhelmed by their understanding of complexities in caregiving, which can impede their ability to remain resilient. Several studies show that better health or mental health literacy is associated with lower caregiver burden and psychological distress, as increased knowledge and skills can improve coping and reduce uncertainty; however, these benefits may depend on the availability of support and resources. In contexts where services are limited, and caregivers feel largely responsible for complex care, greater literacy might increase awareness of gaps in care, risks, and unmet needs, amplifying stress and contributing to lower resilience despite theoretically stronger coping capacities (41–43).

In contrast, the positive correlation between caregiver challenges and resilience denotes a more nuanced relationship. While caregivers facing significant challenges may develop resilience as a coping mechanism, this resilience may not necessarily mitigate the negative impacts of those challenges. Instead, it may reflect an adaptive response to the stressors inherent in caregiving. This observation aligns with the concept that resilience can be both a response to and a product of adversity, indicating that caregivers may draw on their resilience to navigate the difficulties they encounter (42).

The regression analysis of the current study reveals that several socio-demographic factors significantly influence caregiver challenges. Notably, caregiver educational level and employment status are inversely related to the challenges faced. Higher educational attainment equips caregivers with better coping strategies and access to resources, thereby reducing their perceived challenges. This finding aligns with existing literature that reported that caregivers with higher education levels tend to report lower levels of stress and burden (33). In terms of employment status, the analysis categorized caregivers into two groups: employed and unemployed. While employed caregivers face the simultaneous obligations of employment and caregiving, which can increase stress, this research also highlights the possible complexity of balancing these duties. For example, employed caregivers may have less time to devote to caregiving activities, heightening feelings of pressure and stress (43). This dual obligation can complicate their circumstances, especially when compared to individuals who are not employed and thus have more time available for caring chores.

Family monthly income is another critical predictor, with lower income levels correlating with increased challenges for caregivers. Financial strain can limit access to necessary resources and support services, further compounding the stress experienced by caregivers. This is consistent with findings from studies indicating that economic hardship significantly impacts caregiver well-being (37, 44).

Additionally, the age of the older adult receiving care is positively associated with caregiver challenges, indicating that as the care recipient ages, the complexity and demands of caregiving may increase. This aligns with research suggesting that caregivers of older adults often face heightened stress due to the increased likelihood of chronic health issues (45). Although the length of caregiving was not explicitly assessed in this study, it is an important variable to consider for future research. Caregivers who have been in their roles longer may experience different levels of health literacy and resilience, influenced by ongoing exposure to caregiving tasks (43). This might help explain some of the findings, particularly regarding their health literacy and challenges. Caregivers typically accumulate health knowledge over time, gaining insights through personal experience and interactions with healthcare providers.

Health literacy plays a significant role in predicting caregiver challenges. Caregivers with higher health literacy are better equipped to navigate the healthcare system and manage care-related tasks, which can help alleviate some of the stress associated with caregiving (40, 46). Moreover, resilience emerged as a significant predictor of caregiver challenges, indicating that caregivers with greater resilience may face increased challenges rather than decreased ones. This nuanced finding shows that, while resilient caregivers are frequently better at stress management, they may also take on extra caring obligations or emotional loads, posing additional problems. This complexity implies that resilience may encourage caregivers to engage more deeply in caregiving responsibilities, so amplifying their experience of challenges. This finding is crucial, as it suggests that fostering resilience through targeted interventions may help mitigate the adverse effects of caregiving stress, while also prompting considerations on balancing the responsibilities taken on by resilient caregivers. Research has shown that resilience can serve as a protective factor against the adverse effects of caregiving, enabling caregivers to maintain their well-being despite the challenges they face (47).

The mediation analysis indicates a significant negative effect of health literacy on resilience, with a coefficient denoting that higher health literacy is associated with lower levels of resilience. This finding is counterintuitive, as one might expect that increased health literacy would empower caregivers, potentially enhancing their resilience in the face of challenges. However, this result suggests that caregivers with higher health literacy may experience greater awareness of the difficulties associated with caregiving, which could lead to overwhelming stress and thereby reduce their resilience. This aligns with the notion that increased knowledge can sometimes lead to increased anxiety about caregiving responsibilities as caregivers become more acutely aware of the complexities of managing health-related issues (48–50).

In contrast, the effect of resilience on caregiver challenges is not statistically significant. This suggests that resilience, although an important trait, does not directly impact caregivers’ challenges in this context. The lack of significance may indicate that other factors, such as social support or coping strategies, could be more critical in mitigating caregiver challenges than resilience alone. Furthermore, the direct effect of health literacy on caregiver challenges is significant, indicating that higher health literacy is associated with reduced caregiver challenges. This suggests that caregivers who are more informed about health issues may be better equipped to manage their responsibilities, resulting in lower stress levels and fewer challenges (40). The total effect also remains significant, underscoring the importance of health literacy in shaping the caregiving experience. However, the indirect impact through resilience is not statistically significant, as indicated by the bootstrapped confidence interval that includes zero. This reveals that resilience does not mediate the relationship between health literacy and caregiver challenges, indicating that while health literacy directly impacts caregiver challenges, resilience does not serve as a pathway through which this effect occurs.

The moderation analysis explores the role of resilience in the relationship between health literacy and caregiver challenges. The findings indicate that neither health literacy nor resilience significantly predicts caregiver challenges, as evidenced by the coefficients and *p*-values of each variable. Specifically, the negative coefficient for health literacy indicates that higher levels of health literacy are associated with a decrease in caregiver challenges; however, this relationship is not statistically significant. Similarly, resilience does not significantly affect caregiver challenges, indicating that it does not contribute meaningfully to predicting these challenges.

Moreover, the interaction term, which assesses whether resilience moderates the relationship between health literacy and caregiver challenges, is also insignificant. This implies that resilience does not enhance or diminish the impact of health literacy on caregiver challenges. The lack of a significant interaction effect suggests that the relationship between health literacy and caregiver challenges remains consistent across all levels of resilience. This finding is critical as it challenges the assumption that resilience could serve as a protective factor that buffers the adverse effects of low health literacy on caregiver challenges (51).

Despite the overall model being statistically significant, with a notable R^2^ value indicating some explained variance, the interaction effect does not contribute significantly to this variance. This shows that while health literacy may play a role in influencing caregiver challenges, the expected moderating effect of resilience is not supported in this analysis. The results align with previous research highlighting the complexity of caregiver dynamics, indicating that factors beyond resilience may be necessary to understand and mitigate caregiver challenges effectively (52–54).

### Strengths and limitations

This study’s primary strength lies in its focus on an under-researched population: caregivers of older adults with chronic diseases in Egypt, highlighting their unique challenges. Using a cross-sectional descriptive design, following STROBE guidelines, adds rigor. Additionally, validated tools such as the Health Literacy Scale for Family Caregivers, Care Challenge Scale, and Resilience Inventory enhance data reliability. Findings provide valuable insights into the relationships between health literacy, caregiver challenges, and resilience. However, some limitations exist. The cross-sectional design limits causal inferences, and self-reported measures may introduce response bias. Additionally, the study does not consider other factors that influence its findings, such as the diversity of geographical areas, which can limit generalizability. Future research should also focus on personality traits and support systems.

## Conclusion and recommendations

In conclusion, the study highlights the complex interplay between caregiver demographics, health literacy, and the challenges caregivers of older adults face. While caregivers generally exhibit high levels of health literacy and resilience, significant predictors of caregiver challenges include educational level, employment status, family monthly income, the age of the older adult, and the presence of chronic diseases. The analysis reveals a negative correlation between health literacy and caregiver challenges, as well as between health literacy and resilience; however, resilience does not mediate or moderate these relationships. These findings underscore the need for targeted interventions that enhance health literacy and address socioeconomic factors to better support caregivers in managing their responsibilities effectively.

### Nursing implications

The findings of this study have significant implications for nursing, particularly in enhancing the support provided to caregivers of older adults with chronic diseases. Nurses play a critical role in educating caregivers about health literacy, which has been shown to correlate with reduced caregiving challenges. By implementing targeted educational programs that focus on improving health literacy, nurses can empower caregivers with the knowledge and skills necessary to navigate the complexities of managing chronic illnesses. This includes providing transparent and accessible information about disease management, medication adherence, and available resources, which can help alleviate caregiver stress and improve their overall well-being. Interventions such as stress management techniques, coping strategies, and fostering social support networks can enhance caregivers’ resilience, ultimately leading to better caregiving outcomes. It is also essential for nurses to assess the individual circumstances of caregivers, including their educational background and cultural factors that may impact their health literacy and resilience. By adopting a patient-centered approach, nurses can better address the unique challenges faced by each caregiver and provide personalized support. Overall, enhancing health literacy and resilience among caregivers is crucial for improving their caregiving experiences and the quality of care delivered to older adults with chronic diseases.

## Data Availability

The raw data supporting the conclusions of this article will be made available by the authors, without undue reservation.
